# Unraveling the Mechanistic Insights of *Asparagus* Bioactives to Promote Cognitive Health: Insights from the Gut–Brain Axis

**DOI:** 10.3390/nu18183016

**Published:** 2026-09-15

**Authors:** Debayan Das, Somdeb Bose Dasgupta, Samudra Prosad Banik, Debasis Bagchi

**Affiliations:** 1 Department of Bioscience and Biotechnology, Indian Institute of Technology Kharagpur, Kharagpur 721302, West Bengal, India; debayandas014@kgpian.iitkgp.ac.in; 2 Department of Microbiology, Government General Degree College, Narayangarh, Rathipur 721437, West Bengal, India; 3 Department of Biology, College of Arts and Sciences, Adelphi University, Garden City, NY 11530, USA; 4 Department of Pharmaceutical Sciences, College of Pharmacy and Health Sciences, Texas Southern University, Houston, TX 77004, USA

**Keywords:** *Asparagus racemosus*, Shatavarin IV, neuroprotective potential, neuroinflammation, gut microbiota, Gut–Brain Axis

## Abstract

*Asparagus racemosus*, popularly known as Shatavari, is a well-acclaimed medicinal herb for the rejuvenation of reproductive and cognitive health. The therapeutic potential of the herb is primarily due to its famed bioactive compound, Shatavarin IV, which is a steroidal saponin with a close structural resemblance to the female reproductive hormone estrogen. Over the last few decades, independent studies have shown that the steroidal saponins, flavonoids, alkaloids, and polyphenols from the plant can neutralize oxidative stress, arrest neuroinflammation, prevent apoptosis, regulate serotonergic and dopaminergic neurotransmission, and promote synaptic plasticity for holistic cognitive homeostasis. A critical finding in delineating the secret of a healthy brain has been its intricate linkage with a happy gut. Intestinal microbes play a significant role in modulating the synthesis of key neurotransmitters through the diverse plethora of their secreted metabolites, such as short-chain fatty acids. The gut–brain axis, primarily the vagus nerve, acts as a specialized access point for these molecules into the brain. In addition, gut microbes play key roles in ensuring the optimal function of the hypothalamic–pituitary–adrenal axis, the central mechanism for the management and mitigation of physical and emotional stress. Fructooligosaccharide residues, found in abundance in Shatavari, serve as excellent prebiotics for the gut microbial population. This review presents an extensive analysis of the key mechanistic insights obtained thus far on the crossroads of Shatavari with the cells of the nervous system and the microbial community involved therein, including findings from our own laboratory, which could provide long-term solutions in addressing cognitive health.

## 1. Introduction

It is paradoxical that as we progress into the realm of Artificial Intelligence, the epitome of human intelligence, the human brain itself has started to falter from the burdens of stress and disoriented lifestyles. Globally, there has been an exponential increase in the reported cases of cognitive decline as a byproduct of lifestyle diseases, driven by a culmination of insulin resistance, oxidative stress, and neuroinflammation [1], surpassing the threats posed by infections and inherited disorders [2]. In addition, mental-health-related issues have also become common in women with reproductive failures or postpartum depression [3]. In these cases, a complex interplay of multiple intrinsic and extrinsic factors has guided the onset of symptoms. Dietary habits, dominated by refined sugars and saturated fats, have jeopardized the insulin signaling pathway [4], altered redox homeostasis [5], accelerated the deposit of advanced glycation end products (AGEs) [6], and, consequently, elevated the burden of managing misfolded amyloid-like entities [7]. Persistent cravings for food in response to workplace stressors has led to the dampening of autophagy, the primary cellular mechanism for waste disposal and recycling [8], with a simultaneous increase in the concentration of amyloid plaques in the cellular milieu [9].

Mood swings and mental stress, the two characteristic features of women in reproductive or post-reproductive age, are chiefly characterized by a sudden and rapid alteration in the levels of key reproductive hormones, namely estrogen and progesterone [10]. Estrogen is intricately associated with the dopaminergic system and therefore controls crucial cognitive decisions, including reward processing and working memory [11]. Estrogen signaling also promotes the synthesis of key neurotrophins, such as BDNF [12], and thus increases neuronal growth and plasticity. Drops in estrogen levels bring about mood swings by dampening estrogen receptor-mediated signaling in the prefrontal cortex region and hippocampus, which are key areas of the brain aiding in regulating mood, emotion, and memory [13]; in addition, it can cause a sudden spike in the levels of monoamine oxidase, an enzyme responsible for the degradation of neurotransmitters, such as serotonin, dopamine, and norepinephrine [14]. Therefore, it is more than apparent that perturbations in the estrogen signaling pathway are linked to the pathophysiology of cognitive disorders, including those encountered in extreme cases of mental health deterioration. In view of the above discussion, it has become evident that the onset of classical neuropathological syndromes, like Alzheimer’s disease (AD), Parkinson’s disease (PD), and amyotrophic lateral sclerosis (ALS), no longer have their roots exclusively in hereditary factors, as they have evolved to share spaces with lifestyle diseases with the consequent ailing of mental status [1].

The two-way communication between the gut microbiota consortium and the brain, often referred to as the gut–brain axis, is the cornerstone of the Enteric Nervous System (ENS) [15] that controls a plethora of actions, including the functioning of the digestive system, the immune system, and the neuroendocrine system. Therefore, it is also regarded as our second brain [16]. Many studies have independently established that the gut microbiota can both produce and stimulate the synthesis of neurotransmitters, like dopamine, norepinephrine, serotonin, and γ-aminobutyric acid (GABA) [17]. Bacterial metabolites, such as short-chain fatty acids (SCFAs), can pass through the blood–brain barrier, alter microglial activity, and modulate the expression of crucial genes to augment neuronal health [18]. Tryptophan is metabolized by the gut microbiome either to increase serotonin levels [19] or sequester towards the kynurenine pathway to modulate the neuroinflammatory response [20]. In addition, the gut microbiota has also been implicated in the functioning of the hypothalamic–pituitary–adrenal (HPA) axis, the chief neuroendocrine stress effector of the body, through the modulation of cortisol levels. In cases of chronic dysbiosis, elevated cortisol levels result in “leaky gut”, causing the systemic release of lipopolysaccharides and cytokines into the bloodstream and bringing about a state of chronic inflammation [21]. Therefore, it is imperative to state that a healthy flora of gut microbes is integral to cognitive health, and any dysbiosis results in a state of chronic or acute crisis in the central nervous system.

Owing to the multitude of debilitating side effects associated with over-the-counter drugs used for the treatment of dementia and other cognitive ailments, phytotherapeutic formulations have been a traditionally trusted and sought after remedy for neuroinflammatory conditions and holistic rejuvenation of cognitive health [22,23,24,25]. *Bacopa monnieri* (Brahmi), *Withania somnifera* (Ashwagandha), *Centella asiatica* (Gotu Kola), and *Asparagus racemosus* (Shatavari) are some of the more popular medicinal herbs from the Indian subcontinent that have been widely mentioned in both ancient texts and the modern scientific literature for their proven roles in the improvement of cognitive health. In this respect, Shatavari, a perennial herb belonging to the *Asparagaceae* family, was originally known for its management of female reproductive health until around 2010, when a preclinical study showed that it could cure chemically induced amnesia and demonstrated nootropic effects by inhibiting the *acetylcholinesterase* enzyme in specific areas of the CNS [24]. Shatavari contains a rich reservoir of bioactive molecules, including steroidal saponins, flavonoids and alkaloids, with strong antioxidant, adaptogenic, and immunomodulatory effects. In this respect, Shatavarins form the primary and most abundant class of steroidal saponins that constitutes the mainstay of adaptogenic properties of the herb in maintaining endocrine optimization, mitigating stress, and establishing holistic homeostasis [26]. Over the last few decades, many independent studies have demonstrated the potential of bioactives from Shatavarins and other metabolites of the plant. Shatavarins have been found to modulate several key signaling pathways and regulate the expression of vital neurotrophic factors directly involved in the maintenance of synaptic plasticity [27,28,29]. An exciting new impetus in this respect has been added by the observation that Shatavari fibers also serve as excellent prebiotics for the nourishment of key gut microbes, such as *Lactobacilli* and *Bifidobacteria* [25]. In addition, they can arrest “leaky gut” and strengthen the immune system. In spite of the tremendous potential of the plant in rejuvenating neuronal health, the chief bottleneck in the production and mass adoption of Shatavari-based formulations has been standardizing the concentration of active phytochemicals, resulting in dosage uncertainty, as well as a lack of adequate pharmacokinetic profiling. This review presents a cumulative update on the experimentally demonstrated cellular targets of Shatavari, as well as the affirmed or plausible mechanistic insights obtained therein, for the exploration of Shatavari-based therapeutic formulations in boosting cognitive health.

## 2. Prebiotic Nourishment and Gut Barrier Integrity

The human gastrointestinal tract, compared to other regions of the body, is colonized by an abundant and diverse gut microbiota [28], and as such plays integral roles in metabolism, regulation of the immune system, and preservation of intestinal barrier integrity, leading to holistic improvement of host health [29]. The large intestine, especially the colon, contains about 10^10^ to 10^12^ bacterial cells per gram, which are predominantly obligate anaerobes [30]. Therefore, it is apparent that along with the nutrition of the human cells, the associated gut flora also needs a supply of specialized nutrients. In recent years, with the growing global recognition of Ayurveda and advances in gut microbiome research, the term prebiotics has gained considerable attention. Glenn R. Gibson and Marcel Roberfroid in 1995 introduced prebiotics as a non-digestible food ingredient that selectively stimulate the growth and activity of gut microbiota, providing health benefits to the host. They further characterized prebiotics later in 2004 by proposing three essential criteria in which they can: withstand hydrolysis by mammalian digestive enzymes, resist gastric acidity, and avoid absorption in gastrointestinal tract; serve as fermentable substrate for the gut microbiota; and selectively enhance the growth and metabolic activity of beneficial microbial populations associated with host health. Since their introduction in 1995, the concept and definition of prebiotics have evolved considerably with modern advances in gut microbiome research. The International Scientific Association for Probiotics and Prebiotics (ISAPP) redefined the definition of prebiotics in 2016 to describe them as a substrate selectively utilized by host-associated microorganisms that confer health benefits to the host [31]. This updated definition reflects the evolving understanding of host–microbiota symbiotic interactions and underscores the importance of prebiotics as dietary components to maintain gut intestinal homeostasis and promote overall health [32]. Advancements in prebiotic research have substantially improved our understanding of their role in maintaining our health. Beyond their role in the gastrointestinal tract (GIT), prebiotics influence a wide range of physiological process through modulation of the gut-microbiota and their microbial metabolites. However, the molecular mechanisms responsible for each of these physiological effects are yet to be unraveled. Recently, prebiotics are being increasingly incorporated into dietary supplements to boost the gut microbiome, resulting in holistic well-being through the microbiota–host axis [33].

## 3. Role of Shatavari

**Figure 1:**
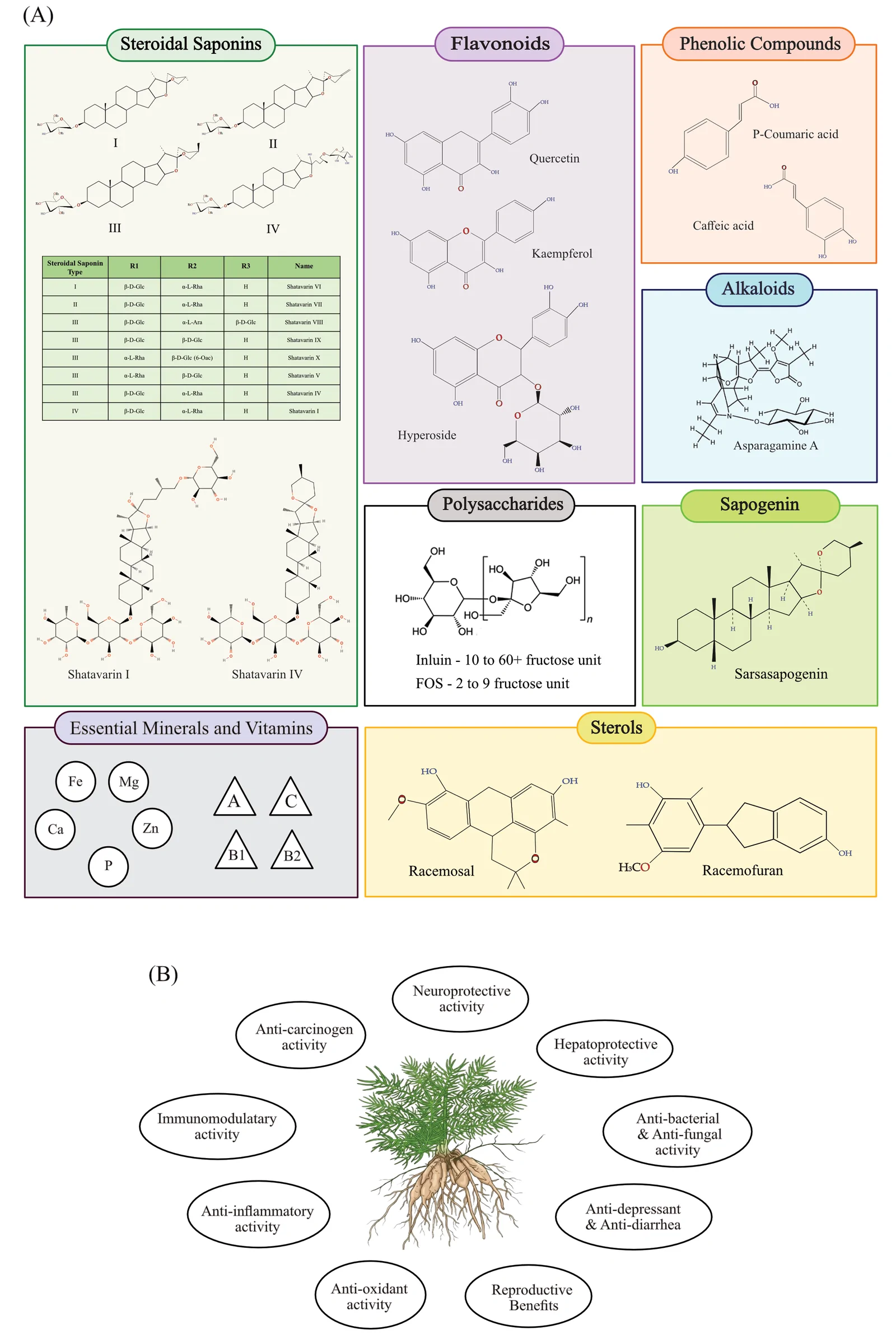
Structure and pharmacological properties of Shatavari. (**A**) Key bioactive components of *Asparagus racemosus* (Shatavari). (**B**) Pharmacological and therapeutic benefits of *Asparagus racemosus* (Shatavari).

Medicinal plants have long been recognized as a rich source of phytochemicals, with diverse bioactive molecules and metabolites that exhibit a wide range of biological effector mechanisms, such as anti-aging, anti-inflammatory, antioxidant, anticoagulant, and neuroprotective activities. In this respect, the role of *Asparagus racemosus* (Shatavari) has gained prominence chiefly due to its abundant reserve of structurally diverse phytochemicals and bioactive metabolites, with a broad spectrum of pharmacological effects and prebiotic potential. The herb contains bioactive constituents, including steroidal saponins (particularly Shatavarins I–IV, sarsasapogenin), flavonoids, polyphenols, alkaloids, and fructooligosaccharides (FOSs) [34] (Figure 1A), which have proven therapeutic benefits (Figure 1B). Among all the steroidal saponins found in Shatavari, Shatavarin IV has attracted particular attention because of its wide range of biological activities followed by its phytoestrogenic properties [35]. Structurally, Shatavarin IV consists of the aglycone steroidal sapogenin, sarsasapogenin, which is linked to one glucose and two rhamnose residues. The aglycone present in Shatavarin IV bears a structural similarity with estrogen. Studies have reported Shatavarin IV can bind to the estrogen receptor and influence the estrogen signaling pathway, hence regulating downstream molecular mechanisms and improving PPD and women’s health [36]. Although Shatavarin IV exhibits a substantially lower affinity for estrogen receptors than the endogenous ligand 17β-estradiol, experimental data indicate that it can modulate both ERα- and ERβ-mediated signaling, resulting in estrogen-like biological effects [37]. Through regulation of estrogen-responsive pathways, Shatavarin IV has been linked to reproductive function [38], increased brain-derived neurotrophic factor (BDNF) expression [39], preservation of synaptic plasticity [40], and modulation of serotonergic and dopaminergic neurotransmissions [41]. These actions are particularly relevant under conditions of estrogen deficiency, such as menopause and postpartum depression, where impaired estrogen signaling contributes to neuroinflammation, mood disturbances, and cognitive decline [28,29]. Collectively, these observations suggest that the phytoestrogenic activity of Shatavarin IV is likely to contribute to the neuroprotective and adaptogenic properties of *Asparagus racemosus* (Shatavari) [42]. In addition to its phytoestrogenic constituents, Shatavari contains fructooligosaccharides (FOSs), which function as fermentable substrates for beneficial members of the gut microbiota, particularly *Bifidobacterium* and *Lactobacillus*. These microorganisms express carbohydrate active enzymes, including fructan hydrolases and glycosidases, which enable the efficient utilization of Shatavari-derived FOSs, a metabolic capability that is largely absent in the host. Preferential fermentation of these oligosaccharides supports the expansion of beneficial commensals while restricting the growth of opportunistic microorganisms through competitive substrate utilization. Fermentation also generates acetate and lactate, which serve as substrates for secondary butyrate-producing bacteria, including *Faecalibacterium*, *Roseburia*, and *Eubacterium* species. This cross-feeding network promotes butyrate production, linking the prebiotic activity of Shatavari to microbial interactions that support intestinal and systemic homeostasis.

Shatavari-derived prebiotics contribute to the restoration of microbial diversity, the stabilization of the intestinal microbial ecosystem, and the recovery of gut homeostasis following dysbiosis. This rejuvenation of beneficial gut commensals forms the basis of many downstream physiological effects associated with Shatavari supplementation [28]. Microbial fermentation of FOSs primarily leads to the generation of short-chain fatty acids (SCFAs). These SCFAs strengthen the intestinal epithelial barrier by serving as an energy source for colonocytes and enhancing the expression of tight-junction proteins, including occludin, claudins, and zonula occludens-1 (ZO-1), thereby reducing intestinal permeability and preventing translocation of microbial endotoxins [43]. SCFAs also suppress the expansion of pathogenic microorganisms by lowering luminal pH and promoting colonization resistance, all while simultaneously limiting the production of pro-inflammatory cytokines and promoting anti-inflammatory immune responses. SCFAs also function as important signaling molecules through the activation of G protein-coupled receptors (GPR41, GPR43, and GPR109A) and the inhibition of histone deacetylases, thereby regulating immune function, cellular metabolism, and gene expression [44]. Further studies have reported that SCFA-mediated signaling influences the microbiota–gut–brain axis by modulating neurotransmitter systems [17]. In particular, SCFAs enhance γ-aminobutyric acid (GABA) production through specific gut microbes and regulate serotonergic signaling by influencing enterochromaffin cell functions and the availability of serotonin precursors [45]. Through these interconnected mechanisms, Shatavari has the potential to restore the gut microbial balance, attenuate inflammation, and promote neurochemical homeostasis, thereby supporting gastrointestinal health alongside emotional and cognitive well-being.

## 4. The Microbiota–Gut–Brain Axis: A Bidirectional Network in Neurodegeneration

**Figure 2:**
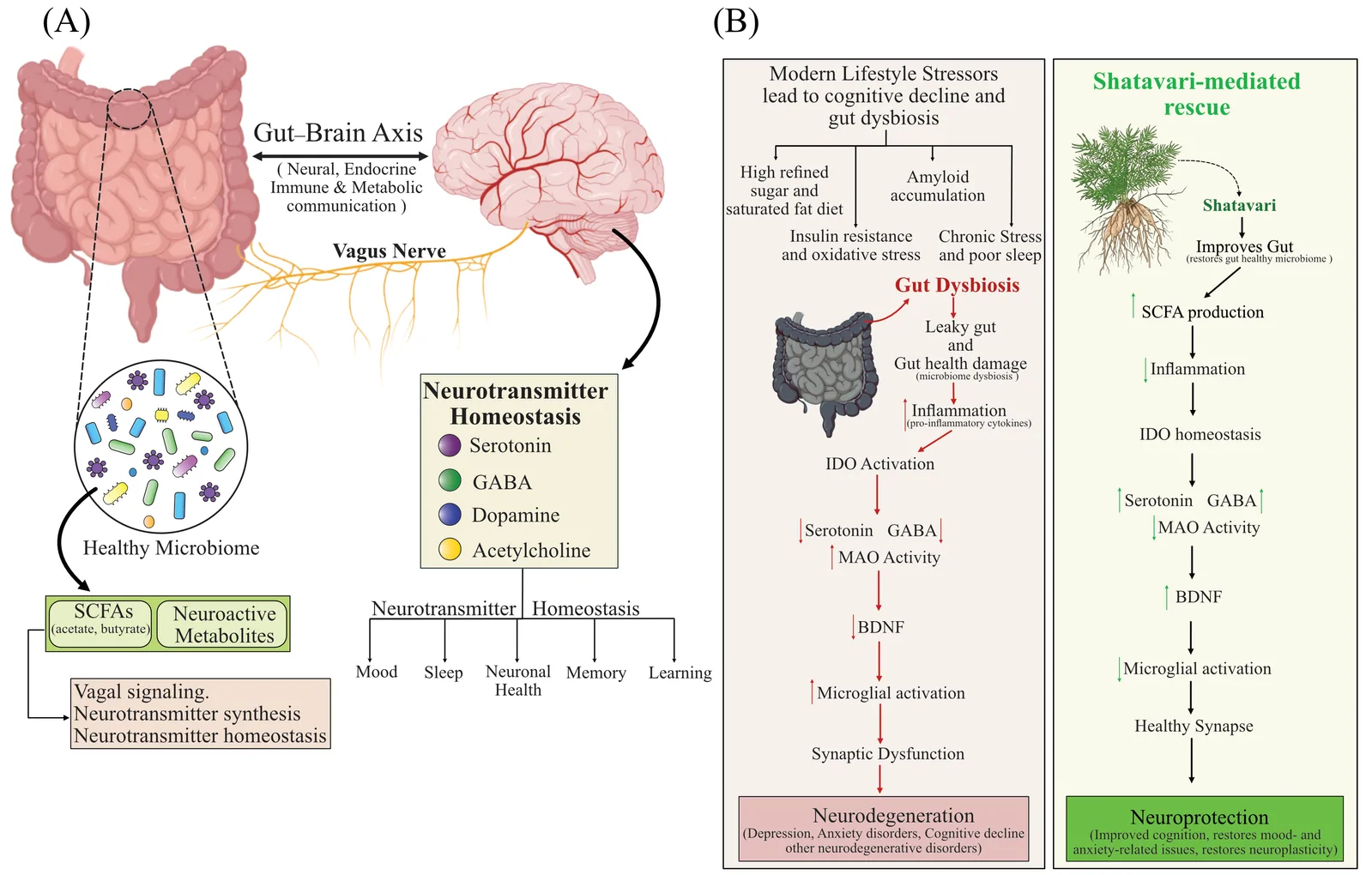
Gut microbiota and the gut–brain axis. (**A**) Schematic representation of a healthy microbiota–gut–brain axis (MGBA), illustrating microbial homeostasis, balanced neurotransmitter signaling, and maintenance of neuronal and systemic health. (**B**) Comparison of gut-dysbiosis-induced alterations in the microbiota–gut–brain axis and their plausible restoration by *Asparagus racemosus* (Shatavari).

The gut–brain axis (GBA) has emerged as a fundamental regulatory network that integrates gastrointestinal physiology with central nervous system functioning through neural, endocrine, immune, and metabolic pathways [17,45,46]. A key component of this communication is the gut microbiota, which regulates brain functions by producing or modulating a variety of neuroactive molecules, including serotonin, γ-aminobutyric acid (GABA), dopamine, and short-chain fatty acids, while simultaneously influencing neuroendocrine signaling, immune responses, and neuronal plasticity (Figure 2A) (Table 1). The microbiota–gut–brain axis (MGBA) is therefore recognized as a bidirectional communication network, where the disruption of gut microbial homeostasis perturbs these signaling networks and contributes to neuroinflammation, oxidative stress, neurotransmitter imbalance, and impaired neuronal function, leading to neuropsychiatric and neurodegenerative disorders [47]. Reciprocally, progressive neurodegeneration disrupts central, autonomic, and neuroendocrine regulations via the ANS (Autonomic Nervous System) and HPA (hypothalamic–pituitary–adrenal) axis, resulting in alteration of gastrointestinal motility, mucus secretion, and barrier integrity. This, in turn, reshapes the gut microenvironment and exacerbates intestinal inflammation, reinforcing disease progression through a self-perpetuating feedback loop [48]. This mutual dependence is evident across functional GI disorders (IBS, FD), metabolic and cardiovascular conditions (obesity, diabetes, hypertension, atherosclerosis), and major neurodegenerative and neuropsychiatric disorders (AD, PD, ASD, depression, anxiety) [46,47], which has shifted the therapeutic paradigm toward interventions that restore gut microbial homeostasis and preserve gut–brain communication [49].

A significant number of recent studies have highlighted the fact that disturbances in gut microbial composition and function, commonly associated with gastrointestinal disorders, such as irritable bowel syndrome (IBS) and functional dyspepsia (FD), as well as systemic disorders, including obesity, diabetes, hypertension, and atherosclerosis, contribute to the development and progression of numerous neuropsychiatric and neurodegenerative disorders [17,46,48]. In reciprocation, the increasing global prevalence of neurodegenerative disorders, including Alzheimer’s disease (AD) and Parkinson’s disease (PD), together with neuropsychiatric disorders, such as depression, anxiety disorder, and autism spectrum disorder (ASD), further highlight the bidirectional nature of the microbiota–gut–brain axis. Progressive neurological dysfunction disrupts autonomic and neuroendocrine regulation, primarily through the autonomic nervous system (ANS) and hypothalamic–pituitary–adrenal (HPA) axis, resulting in altered gastrointestinal motility, impaired mucus secretion, compromised intestinal barrier integrity, dysregulated mucosal immune responses, and changes in nutrient availability within the intestinal lumen. These alterations reshape the gut microenvironment, promote microbial dysbiosis, and exacerbate intestinal inflammation, thereby establishing a self-perpetuating microbiota–gut–brain feedback loop that reinforces disease progression. Recognition of this reciprocal interaction has shifted the therapeutic paradigm toward therapeutic interventions that restore gut microbial homeostasis and preserve microbiota–gut–brain communication, offering promising opportunities to attenuate neurodegeneration [78].

As a result, strategies aimed at restoring gut microbial homeostasis are increasingly being explored and investigated for their potential to mitigate these pathological alterations and improve cognitive and emotional health to further prevent or slow down the progression of neurodegenerative disorders [47,49]. In this context, growing scientific interest has been directed towards naturally derived bioactive compounds, particularly those used in traditional medicines, such as Ayurveda. Although Ayurvedic formulations have been employed for centuries to promote physical and mental well-being, recent advances in molecular biology, immunology, neurobiology, metabolomic, and microbiome research have enabled the mechanistic understanding of both their pharmacological and pathophysiological effects. Over the past decade, increasing attention has been directed towards understanding how herbal bioactive compounds influence the microbiota–gut–brain axis and contribute to neuronal health. Despite these advances, the molecular mechanisms linking individual phytochemicals to their cellular targets and downstream signaling pathways remain largely undeciphered. Integrating traditional Ayurvedic knowledge with contemporary molecular research has renewed interest in medicinal plants as modulators of gut–brain communication. Among these, *Asparagus racemosus* (Shatavari) is distinguished by its diverse phytochemical composition and reported antioxidant, anti-inflammatory, immunomodulatory, neuroprotective, and prebiotic activities. The current evidence indicates that these properties influence multiple pathways involved in gut physiology, immune regulation, and neuronal function, supporting the potential of Shatavari to improve cognitive health through the modulation of the microbiota–gut–brain axis [25] (Figure 2B).

## 5. Neurotransmitter Regulation and Cognitive Function

Neurotransmitters are chemical messengers that mediate interneuronal communication, as well as communication between neurons and their effector cells. Precise regulation of neurotransmitter synthesis, vesicular release, receptor activation, reuptake, and enzymatic degradation is essential for maintaining neuronal signaling, cognitive function, and brain homeostasis [79]. Increasing evidence indicates that the gut microbiota contributes to these regulatory processes by influencing the availability of neurotransmitter precursors, generating neuroactive metabolites, and producing compounds capable of modulating neuronal signaling. Consequently, alterations in the gut microbial composition can disrupt the neurotransmitter balance and have been associated with depression, cognitive impairment, and several neurodegenerative disorders [47].

Many intestinal microorganisms participate directly in neurotransmitter metabolism. Species belonging to the genera *Lactobacillus*, *Bifidobacterium*, *Enterococcus*, and *Streptococcus* produce neuroactive molecules, including γ-aminobutyric acid (GABA), serotonin, dopamine, and acetylcholine [17]. Gut microbes also generate short-chain fatty acids (SCFAs), particularly acetate, propionate, and butyrate, which can influence enterochromaffin cell functions, maintain blood–brain barrier integrity, regulate microglial maturation, and modulate neuroimmune signaling. Through these complementary mechanisms, the gut microbiota serves as an important regulator of neurotransmission within the microbiota–gut–brain axis [80]. Thus, alterations in microbial composition can profoundly affect neurotransmitter homeostasis and consequently influence neuronal responses [48]. Gut dysbiosis induces alterations in neurotransmitter signaling, leading to depression, anxiety, cognitive decline, Alzheimer’s disease, Parkinson’s disease, and other neurodegenerative conditions. Therefore, the restoration of neurotransmitter balance constitutes a critical therapeutic strategy for maintaining neuronal and cognitive resilience [46]. Among the various neurotransmitter systems involved in microbiota–gut–brain axis (MGBA) communication, serotonergic and GABAergic signaling have emerged as major regulatory pathways governing cognition, mood, learning, memory, and emotional processing. In addition, the modulation of monoamine oxidase (MAO) activity has gained increasing attention because of its role in maintaining monoaminergic neurotransmitter homeostasis. Given the growing evidence that *Asparagus racemosus* (Shatavari) modulates these pathways, this review primarily focuses on the interplay between serotonergic signaling, GABAergic neurotransmission, and MAO inhibition in mediating the neuroprotective and microbiota-dependent effects of Shatavari. Shatavari influences the synthesis and preservation of key mood-regulating mechanisms, like serotonin synthesis, GABAergic support, and MAO inhibition [48].

### 5.1. Shatavari in Maintaining Serotonergic Homeostasis in the MGBA

Serotonin, or 5-hydroxytryptamine (5-HT), is a monoamine metabolite derived from dietary tryptophan. It is a crucial neurotransmitter of both the peripheral nervous system (PNS) and the central nervous system (CNS), and it also happens to be one of the principal biochemical mediators of the MGBA, facilitating bidirectional communication between the gastrointestinal tract (GIT) and CNS. Approximately 90% of the body’s total serotonin reserve is synthesized and stored in the gastrointestinal tract by a group of specialized cells known as the enterochromaffin cells (ECs) and is under the regulation of the peripheral nervous system (PNS), while the remaining 10% is synthesized in the brain stem (CNS) by a specialized group of neurons called serotonergic neurons (SNs). The serotonergic pathway has received particular attention due to its central role in cognitive behaviors, like mood regulation, learning, memory formation, emotional processing, and neuroplasticity [81]. The gut and brain continuously exchange neural, endocrine, immune, and metabolic signals, with serotonin acting as an important proctor of these physiological processes. Serotonergic signaling maintains gut–brain homeostasis through coordinated regulation of gastrointestinal motility, vagal signaling, neuroendocrine responses, and central neurotransmission.

Serotonin biosynthesis is a two-step process consisting of hydroxylation and decarboxylation. The former is initiated by the rate-limiting enzyme tryptophan hydroxylase (TPH), followed by decarboxylation to L-amino acids by decarboxylase. Two TPH isoforms account for the spatial difference in serotonin synthesis. TPH1 is expressed in enterochromaffin cells (ECs) of the intestinal epithelium and in other non-neuronal peripheral tissues. This isoform predominantly regulates peripheral serotonin production within enterochromaffin cells produced by the gut. The other isoform, TPH2, is expressed in serotonergic neurons of the brain stem raphe nuclei and in a subset of enteric neurons [82]. It governs the neuronal serotonin within the raphe nuclei of the CNS [83]. Intestinal ECs account for roughly 90–95% of the total body serotonin [84,85]. Only a small fraction, approximately 1–2%, resides in the CNS. Both isoforms are therefore present in the gut wall, where a large non-neuronal EC pool sits, alongside smaller contributions from enteric neurons and mucosal mast cells [86].

The two serotonin pools do not exchange. Serotonin cannot cross the blood–brain barrier, so 5-HT produced in the gut does not enter the brain, and central 5-HT makes no measurable contribution to the circulating concentrations [85]. Gut serotonin regulates fluid levels and powers peristaltic movement, lubricating the bolus and ensuring the smooth and rhythmic movement, thus helping in proper digestion and nutrient uptake [85]. The gut dumps massive amounts of serotonin when it detects bad food and bacteria, forcing the intestine to flood the colon with water and cause diarrhea. An imbalance of the gut serotonin homeostasis causes chronic diarrhea and severe constipation, leading to irritable bowel syndrome, like IBS-D, IBS-C, IBS-M, and IBS-U. In the CNS, serotonin is the master emotional rheostat. It governs mood and emotion, dictates sleep–wake cycles, acts as a gatekeeper for pain signals, and manages cognitive function and the brain’s reward and attention pathways [87]. Serotonin triggers the release of brain-derived neurotrophic factor (BDNF), a neurotrophin essential for neuronal differentiation, synaptic remodeling, neuroplasticity, and neuronal survival. Impairment of serotonergic signaling and reduced BDNF expression impairs cognitive function and learning, damages synaptic plasticity, and reduces neuroprotection, thereby leading to neurodegenerative disorders [88]. Monoamine oxidase degrades 5-HT to 5-hydroxyindoleacetic acid (5-HIAA) in both compartments, connecting the serotonergic tone to MAO-directed mechanisms, which will be discussed later in Section 5.3.

Although serotonin is synthesized by the host cells in different locations, the gut microbiota plays an essential role in their production and availability. Under physiological conditions, commensal microbial populations facilitate balanced tryptophan metabolism, allowing for an appropriate balance between serotonin biosynthesis and kynurenine metabolism while supporting normal serotonergic signaling [89]. However, the microbiota acts on these two pools by different routes. Its effect on the peripheral pool is direct; germ-free mice have previously shown markedly reduced colonic and serum 5-HT, and colonization with indigenous spore-forming bacteria raised colonic TPH1 expression together with mucosal, luminal, and platelet serotonin [86]. Its effect on the central pool is indirect and less settled. Germ-free male mice carry elevated plasma tryptophan and elevated hippocampal 5-HT and 5-HIAA, and post-weaning colonization restored tryptophan availability without restoring the hippocampal changes [90], which points to a developmental window rather than a simple dose–response relationship between microbial load and central serotonin.

Tryptophan partitioning is the shared control point. Under physiological conditions, commensal populations support a balance between serotonin biosynthesis and kynurenine metabolism [85]. Dysbiosis and persistent inflammation shift that balance by inducing indoleamine 2,3-dioxygenase (IDO), the rate-limiting enzyme that channels tryptophan toward the kynurenine pathway [81]. As less precursor reaches both TPH isoforms, the profile of kynurenine metabolites are also altered concurrently.. Quinolinic acid acts as an *N*-methyl-D-aspartate (NMDA) receptor agonist promoting excitotoxicity, oxidative stress, and neuronal damage, whereas kynurenic acid antagonizes excitatory neurotransmission and is neuroprotective. Sustained activation of this pathway therefore forms a molecular interface through which chronic inflammation influences neurotransmitter homeostasis, and it has been linked to depressive disorders, impaired cognitive performance, and several neurodegenerative diseases [91]. Species-level evidence supports this partitioning role. Bacterial species like *Streptococcus*, *Bifidobacterium*, *Lactobacillus*, *Klebsiella*, and *Escherichia coli* have all been reported to modulate serotonin homeostasis by modulating tryptophan metabolism [92]. Animal model studies have revealed significant insights regarding how specific microbes aid in altering systemic tryptophan and serotonergic activities and hence act at different points of the pathway [93]. Fourteen days of *Bifidobacterium infantis* in Sprague-Dawley rats attenuated IFN-γ, TNF-α, and IL-6 responses to mitogen stimulation, raised plasma tryptophan and kynurenic acid, and lowered 5-HIAA in the frontal cortex, indicating a reduced central serotonin turnover, although the swim behavior in naive animals was unchanged [94]. *Lactobacillus johnsonii* fed to BioBreeding diabetes-prone rats lowered serum kynurenine by 17% with a 1.4-fold rise in 5-HT, an effect traced to hydrogen peroxide inhibition of intestinal IDO [95]. These results place most of the microbial contributions upstream of serotonin itself in the partitioning of tryptophan between the two competing routes.

Insults to the gut and CNS resulting from factors, such as an unhealthy diet, chronic psychological stress, antibiotic overuse, gastrointestinal infections, aging, environmental toxins, alcohol consumption, smoking, sleep disturbances, and pathological protein aggregation (e.g., amyloid-β and tau in neurodegenerative diseases), can disrupt microbiota–gut–brain axis homeostasis, leading to alterations in gut microbial composition, impaired intestinal barrier function, chronic neuroinflammation, and dysregulated serotonergic signaling. As discussed earlier, Shatavari possesses prebiotic properties; therefore, the growth of beneficial bacterial genera, particularly *Lactobacillus* and *Bifidobacterium*, are associated with the maintenance of gut microbial homeostasis [25] through which Shatavari indirectly regulates neurotransmitter homeostasis along the gut–brain axis [96].

Shatavari may help maintain serotonergic homeostasis by supporting intestinal health and limiting inflammatory signaling. The mechanism proposed here is indirect and acts on the central pool. However, by supporting barrier integrity and limiting inflammatory signaling, Shatavari would be expected to reduce IDO induction and preserve the fraction of tryptophan available for transport into the brain, instead of acting on serotonin synthesis directly [28]. The central serotonergic tone is, in turn, associated with mood regulation, stress resilience, synaptic plasticity, and cognitive function. No study has yet tested this prediction for *A. racemosus*, and it should be read as a hypothesis consistent with the prebiotic and anti-inflammatory data rather than as a demonstrated mechanism.

### 5.2. Shatavari in Maintaining GABAergic Homeostasis in the MGBA

Gamma-aminobutyric acid (GABA) is the principal inhibitory, non-proteinogenic neurotransmitter, whereas glutamate serves as the major excitatory neurotransmitter in the central nervous system (CNS). Together, these neurotransmitters function as complementary regulators of neuronal activity, and their coordinated actions maintain the excitatory–inhibitory balance required for efficient synaptic transmission and normal brain function. This balance is fundamental for neuronal excitability, neural circuit maturation, cognitive processes, and regulation of the sleep–wake cycle. Disturbances in GABAergic or glutamatergic signaling can disrupt this equilibrium, resulting in neuronal hyperexcitability or defective synaptic communication, both of which have been implicated in the development of several neurological and neuropsychiatric disorders [97].

Glutamate is the brain’s primary excitatory neurotransmitter and a key precursor metabolite for the synthesis of GABA. Glutamate, produced from the glycolytic flux, is converted into GABA by the enzyme glutamate decarboxylase (GAD), the rate-limiting step in GABA synthesis. Pyridoxal-5′-phosphate (PLP), the active form of Vitamin B_6_, is the co-factor for GAD. Newly synthesized GABA is packed into synaptic vesicles by the vesicular GABA transporter (VGAT). Neuronal depolarization triggers the opening of voltage-gated calcium channels in the presynaptic neuron, resulting in Ca^2+^ influx. Increasing the intracellular calcium concentration induces the fusion of GABA-containing synaptic vesicles with the presynaptic neuron membrane, thereby leading to the exocytotic release of GABA into the synaptic cleft. The GABAergic signal is terminated through the rapid uptake of GABA from the synaptic cleft into the presynaptic neurons and surrounding astrocytes by GABA transporters (GATs). Following reuptake, GABA is catabolized by GABA transaminase (GABA-T) into succinic semialdehyde, which is further converted to succinate by succinic-semialdehyde dehydrogenase (SSADH). Succinate further re-enters the tricarboxylic cycle (TCA), linking GABA metabolism with cellular energy production. This metabolic pathway is known as the GABA shunt, and it provides an alternative pathway that integrates neurotransmitter metabolism with mitochondrial energy generation while contributing to the maintenance of neuronal metabolic homeostasis [98].

In addition to host metabolism, the gut microbiota consortium is well capable of producing GABA or modulating host GABA metabolism, emphasizing the intimate relationship between the gut microbiome and neurotransmitters [17]. *Asparagus racemous* contains flavonoids and phenolic compounds that have been reported to influence GABAergic signaling and exhibit anxiolytic activity [25]. It can further enhance inhibitory neurotransmission, attenuate stress-induced neuronal hyperexcitability, and promote emotional homeostasis [99]. The preservation of GABAergic functions may also create a favorable neurochemical environment for synaptic plasticity, thereby supporting learning, memory formation, and cognitive flexibility.

### 5.3. Shatavari and MAO Inhibition

Monoamine oxidases (MAO-A and MAO-B) catalyze the oxidative deamination of serotonin, dopamine, norepinephrine, and other monoamine neurotransmitters, regulating their intracellular and synaptic concentrations [14]. Increased MAO activity reduces monoamine availability and, in turn, accelerates neurotransmitter depletion and cognitive dysfunction, thus further contributing to mood and other age-related neurodegenerative disorders [100]. Phytochemical studies of Shatavari have also reported that its steroidal saponins, particularly Shatavarin IV, inhibit monoamine oxidase activity, thereby suggesting a possible mechanism through which Shatavari could prolong neurotransmitter availability and enhance synaptic signaling [42].

The MGBA coordinates the activity of the serotonergic, dopaminergic, GABAergic, and cholinergic systems to regulate cognition, emotion, learning, memory, and gastrointestinal physiology [101]. Emerging evidence suggests that Shatavari may indirectly modulate the MGBA by promoting the growth and persistence of beneficial gut microorganisms [25], thereby restoring the gut microbial niche and improving microbial homeostasis. Through its prebiotic phytoconstituents, Shatavari may support intestinal barrier integrity, attenuate inflammation, and preserve neurotransmitter precursor metabolism, collectively contributing to the maintenance of neurotransmitter homeostasis. Although the underlying molecular mechanisms require further investigation, restoration of gut microbial homeostasis by Shatavari may help mitigate microbiota-associated dysfunctions implicated in gastrointestinal disorders, including irritable bowel syndrome (IBS), as well as neuropsychiatric and neurodegenerative disorders, such as anxiety, stress-related disorders, Alzheimer’s disease, Parkinson’s disease, and Huntington’s disease.

## 6. Modulation of the HPA Axis and Neuroinflammation

### 6.1. Shatavari and HPA Axis

**Figure 3:**
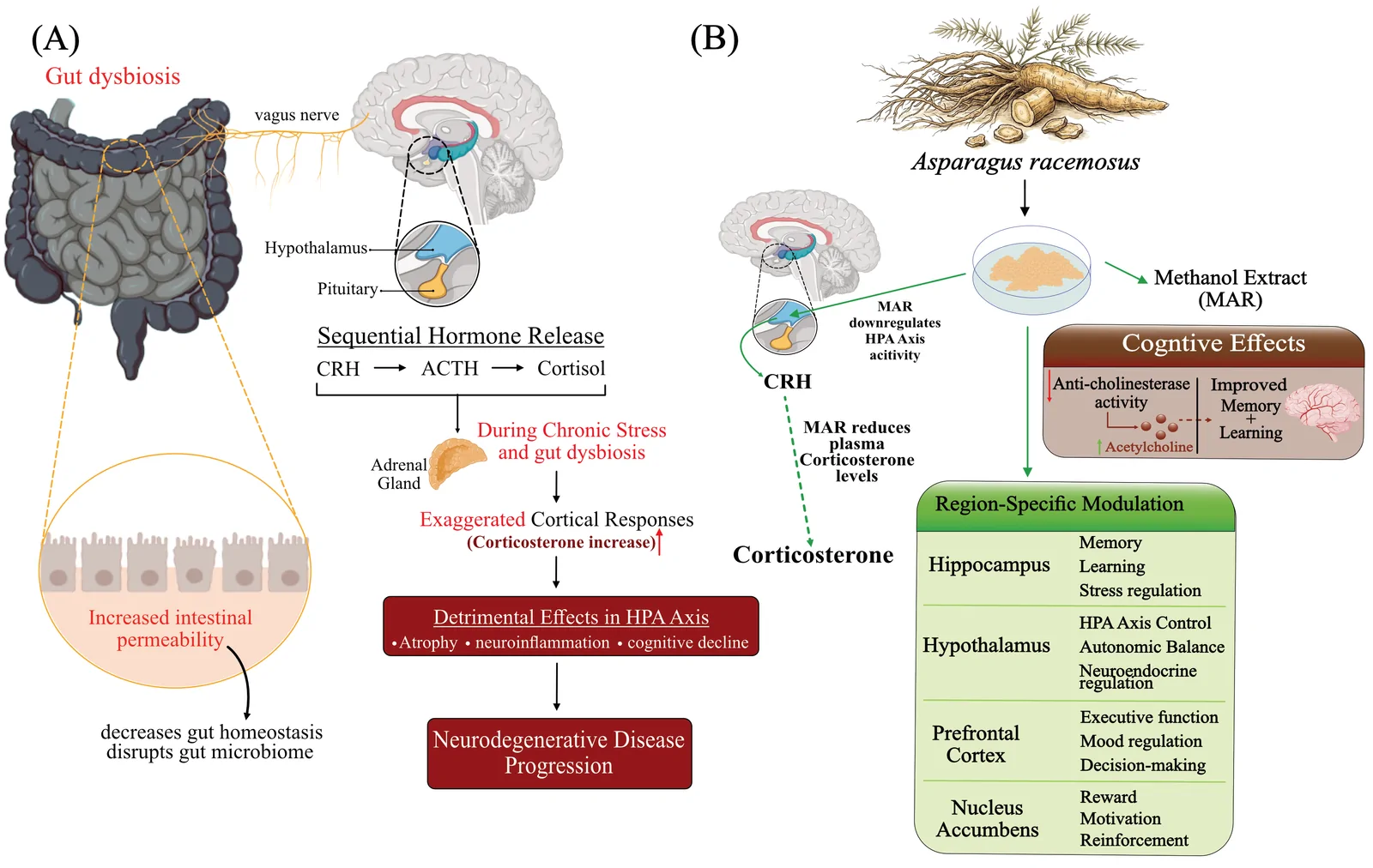
Shatavari-mediated amelioration of gut-dysbiosis-affected HPA axis. (**A**) Dysregulation of the hypothalamic–pituitary–adrenal (HPA) axis during gut dysbiosis. (**B**) Proposed restoration of HPA axis function, region-specific neuromodulation, and cognitive improvement following treatment with methanolic extract of *Asparagus racemosus* (MAR).

The hypothalamic–pituitary–adrenal (HPA) axis represents one of the principal neuroendocrine components of the gut–brain axis through which intestinal disturbances influence brain functions. The HPA axis serves as the central coordinator of physiological responses to stress and plays a critical role in maintaining systemic homeostasis. Communication between the gut microbiota and the HPA axis is also bidirectional. Psychological stress alters the gut’s microbial composition, whereas gut dysbiosis modulates neuroendocrine responses through immune, neural, and metabolic signaling pathways. As a result, chronic dysregulation of the HPA axis contributes significantly to depression, cognitive impairment, and neurodegenerative progression [102] (Figure 3A).

Studies have suggested that gut dysbiosis disrupts intestinal barrier integrity, leading to increased intestinal permeability and translocation of microbial products, such as lipopolysaccharide (LPS), into the systemic circulation. Circulating LPS binds to toll-like receptor 4 (TLR4) expressed on the macrophages, dendritic cells, and other innate immune cells, initiating MyD88-dependent intracellular signaling that activates both the NF-κB and MAPK pathways. This signaling cascade leads to the secretion of pro-inflammatory cytokines TNF-α, IL-6, and IL-1β. These cytokines subsequently activate hypothalamic stress circuits, stimulating the sequential release of corticotropin-releasing hormone (CRH), adrenocorticotropic hormone (ACTH), and cortisol [60].

A transient increase in cortisol levels facilitates the adaptation to stress; however, persistent activation of the HPA axis results in prolonged elevations of the cortisol levels, causing detrimental effects on neuronal health, impairing hippocampal neurogenesis, reducing synaptic plasticity, suppressing neurotrophic signaling, and accelerating cognitive decline [103]. Clinical studies have reported HPA axis dysregulation has been associated with depression, anxiety disorders, neuroinflammation, and neurodegenerative diseases. These observations highlight the importance of HPA homeostasis and suggest that the potential restoration of HPA axis homeostasis could be an important therapeutic strategy for preserving cognitive function [104]. Adaptogens are natural compounds that enhance resistance to physiological and psychological stressors by improving stress tolerance and promoting homeostasis through modulation of neuroendocrine and immune responses. Among the medicinal plants with documented adaptogenic properties, *Asparagus racemosus* (Shatavari) has been extensively used in traditional medicine for its ability to enhance resilience against physical, emotional, and metabolic stress [42].

In addition to neuroendocrine dysregulation, chronic activation of the HPA axis is closely intertwined with neuroinflammatory processes that further exacerbate neuronal dysfunction and cognitive decline [105]. Therefore, therapeutic strategies capable of simultaneously modulating stress responses and inflammation have attracted considerable interest. As a well-established adaptogenic herb, Shatavari has been reported to stabilize HPA axis activity, attenuate exaggerated cortisol responses, and improve resilience against physical, emotional, and metabolic stress [99]. Adaptogens are also believed to reduce oxidative stress and inflammation-induced neuronal dysfunction in addition to regulating neuroendocrine function [106]. Collectively, these observations suggest that Shatavari protects vulnerable neuronal populations from chronic glucocorticoid exposure while preserving cognitive function [40,101]. Properties from Shatavari are particularly relevant in conditions such as postpartum depression, where endocrine fluctuations coincide with heightened stress sensitivity [25].

### 6.2. Shatavari and Neuroinflammation

Neuroinflammation is another key pathophysiological mechanism linking gut dysfunction to cognitive impairment [107]. Elevated concentrations of pro-inflammatory cytokines, such as TNF-α, IL-6, and IL-1β, have consistently been associated with depression, neurodegeneration, and impaired cognitive performance [60]. These inflammatory mediators interfere with neurotransmitter homeostasis, disrupt neuronal signaling, compromise synaptic plasticity, and promote oxidative damage [64]. Peripheral inflammatory cytokines influence brain functions by activating microglia, the resident immune cells of the central nervous system. Under physiological conditions, microglia contribute to immune surveillance and maintenance of neuronal homeostasis. However, chronic exposure to inflammatory mediators promotes sustained microglial activation, resulting in the excessive production of TNF-α, IL-6, IL-1β, reactive oxygen species, and other neurotoxic mediators. The sustained release of these mediators establishes a chronic neuroinflammatory environment that compromises synaptic plasticity, impairs neuronal survival, and accelerates progression of neurodegenerative disorders, including Alzheimer’s disease and Parkinson’s disease [108]. Experimental evidence has demonstrated the anti-inflammatory potential of Shatavari. Its bioactive constituents have been reported to suppress the production of major pro-inflammatory cytokines while attenuating multiple inflammatory signaling cascades [109] (Table 2). Given that inflammatory cytokines are major inducers of IDO activity, the attenuation of inflammatory signaling may also limit excessive activation of the kynurenine pathway, thereby preserving tryptophan availability for serotonin biosynthesis while reducing the accumulation of neurotoxic kynurenine metabolites [110]. Computational docking analyses have further suggested that the bioactive component from Shatavari, Shatavarin IV, possesses substantial binding affinity toward TNF-α, raising the possibility that it may directly interfere with cytokine-mediated inflammatory responses. Shatavari appears to suppress neuroimmune activation, interrupting the self-perpetuating cycle that links inflammation, oxidative stress, neurotransmitter imbalance, and cognitive dysfunction [29,40,41,111].

The anti-inflammatory properties of Shatavari are further supported by its ability to regulate intracellular inflammatory signaling pathways [27]. The nuclear factor-kappa B (NF-κB) and mitogen-activated protein kinase (MAPK) pathways represent major regulators of cytokine production and microglial activation. Persistent activation of these pathways promotes a pro-inflammatory microenvironment within the central nervous system, resulting in sustained neuronal injury and progressive neurodegeneration [118]. Flavonoids and phenolic compounds present in Shatavari have been reported to suppress NF-κB and MAPK signaling, thereby limiting inflammatory amplification and preserving neuronal homeostasis [119]. Since NF-κB and MAPK signaling also regulate microglial activation, suppression of these pathways appears to interrupt the feed-forward inflammatory cycle linking gut dysbiosis with neurodegeneration.

In addition to transcriptional regulators of inflammation, several intracellular kinases participate in inflammatory signaling and cause neuronal degeneration. Among these, glycogen synthase kinase-3β (GSK-3β) has emerged as a critical molecular hub owing to its central role in neuroinflammation, synaptic dysfunction, tau hyperphosphorylation, and amyloid-β pathology [120]. Dysregulated GSK-3β activity has been implicated in Alzheimer’s disease, depression, and several other neurodegenerative disorders. In silico docking studies have indicated that Shatavarin IV exhibits significant binding affinity toward GSK-3β and the GSK-3β–Axin complex [102], suggesting a potential molecular interaction. Docking affinity alone, however, does not establish the functional inhibition of GSK-3β activity; these predictions reflect a theoretical binding potential rather than confirmed enzymatic or cellular effects. If experimentally validated, this interaction could represent one of the key mechanisms by which Shatavari modulates neuroinflammation, amyloid-β deposition, and neurofibrillary tangle formation. In parallel, biochemical assays, together with cellular studies, are the other mandates by which the role of Shatavarin IV can be confirmed in the functional inhibition of GSK-3β, thereby establishing this pathway as a genuine contributor to the neuroprotective effects of *Asparagus racemosus* (Shatavari). It is equally worthwhile to consider whether Shatavarin IV’s concentration realized in the brain microenvironment is sufficient to produce these predicted effects. The evidence gathered so far in SH-SY5Y cell lines, *C. elegans* models, and the docking analysis as mentioned above hints at a plausible mechanism, but none of these show that Shatavarin IV or its metabolites cross the blood–brain barrier or accumulate at active concentrations in the mammalian CNS. Nanoemulsion-based formulations of Shatavarin IV [121] have been explored for Alzheimer’s disease applications and represent one route toward improving CNS delivery, though this would still need confirmation through in vivo pharmacokinetic studies measuring actual brain-tissue concentrations.

## 7. Oxidative Stress, Neuroplasticity, and Cognitive Resilience

Oxidative stress represents a common pathological root cause in the etiology and pathogenesis of neurological disorders and cognitive impairments. Increased free-radical production (ROS) disrupts cellular redox homeostasis, leading to oxidative stress and the onset of neuronal degeneration and disorders. ROS generation during oxidative stress damages cellular lipids, proteins, and nucleic acids, compromising neuronal survival and synaptic integrity. Neurons are more vulnerable to oxidative injury due to their high metabolic activity and substantial oxygen consumption. Studies have revealed that oxidative stress leads to the pathogenesis of many neurodegenerative diseases, including Alzheimer’s disease (AD), mild cognitive impairment (MCI), Parkinson’s disease (PD), Huntington’s disease (HD), amyotrophic lateral sclerosis (ALS), and several others [122].

**Figure 4:**
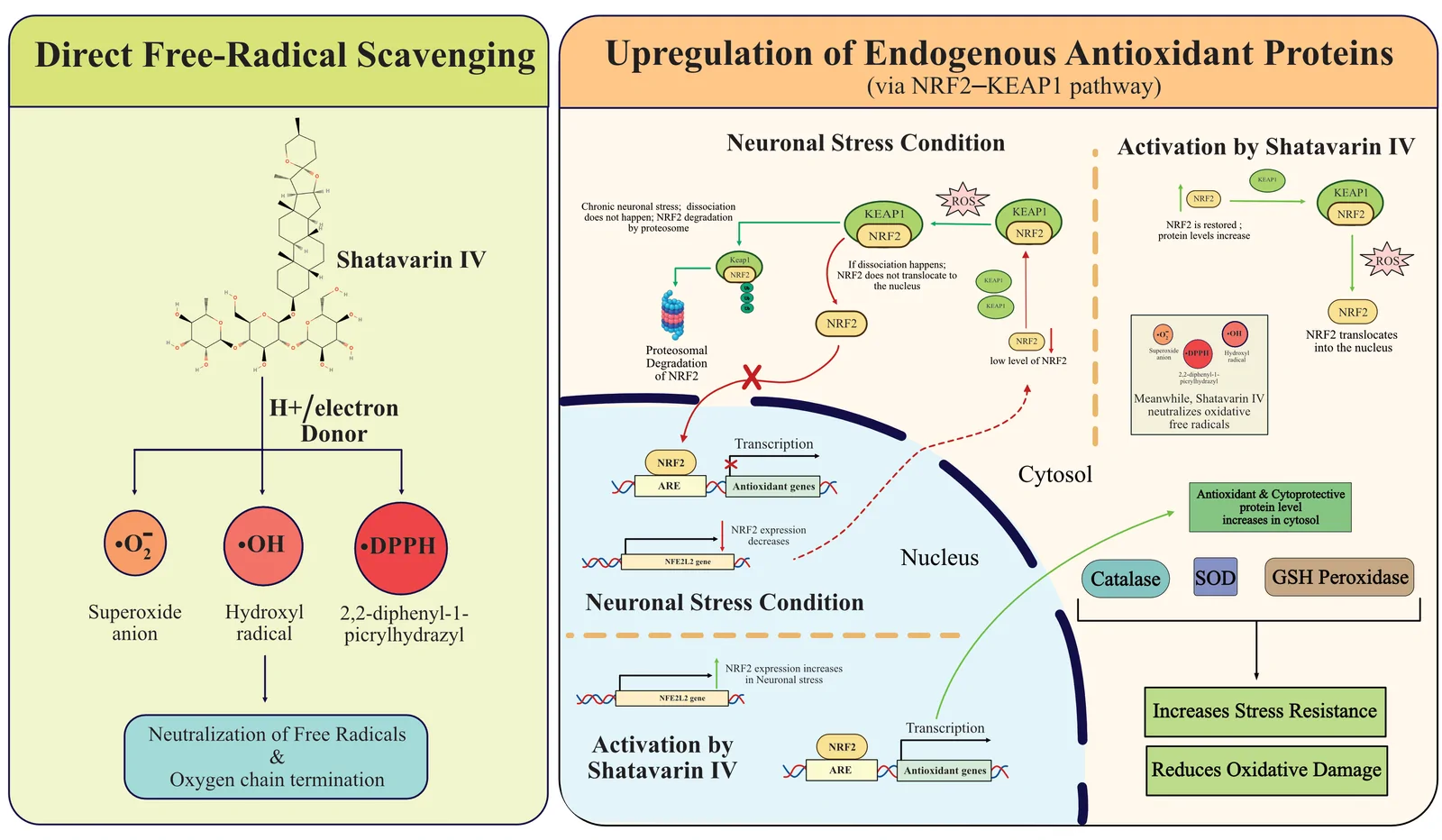
Shatavari-mediated proposed restoration of inflammation-induced damage response pathway in the CNS: NRF2 under normal conditions is maintained at a low level through proteasomal activity via the NRF2-KEAP1 pathway. In the cytosol, NRF2 is sequestered by KEAP1, leading to ubiquitination followed by proteasomal degradation. When ROS is triggered, NRF2 is released from KEAP1, translocates to the nucleus, binds to ARE gene sequences, and activates antioxidant genes, like SOD, catalase, GSH peroxidase, etc. Under chronic neuronal stress, this pathway is hampered by a hitherto unknown mechanism, leading to a low expression of NRF2 levels that fail to associate with KEAP1. Shatavarin IV plausibly revives this translocation, as reflected by the increase in levels of SOD2, catalase, and GSH peroxidase (green arrows indicate experimentally verified and known pathways; red arrows indicate hypothesized pathways for which no direct experimental evidence is available).

In order to mitigate the threat posed by oxidative stress, cells rely on the nuclear factor erythroid 2-related factor 2 (NRF2) signaling pathway, one of the major regulators of the endogenous antioxidant response. Under normal physiological conditions, NRF2 remains sequestered in the cytoplasm by Kelch-like ECH-associated protein 1 (KEAP1), and the sequestered NRF2 is continuously targeted for proteasomal degradation by KEAP1-mediated ubiquitination. During oxidative stress, NRF2 dissociates from KEAP1 and translocates to the nucleus, where it binds to antioxidant response elements (AREs) to induce the transcription of cytoprotective genes, including heme oxygenase-1 (HO-1), NAD(P)H quinone oxidoreductase-1 (NQO1), superoxide dismutase (SOD), catalase, and other enzymes involved in glutathione synthesis and recycling. Activation of this pathway restores intracellular redox homeostasis, preserves mitochondrial integrity, and attenuates ROS-mediated cellular injury [123] (Figure 4).

Shatavari possesses potent antioxidant properties and enhances endogenous antioxidant defense systems. While direct mechanistic studies remain limited, Shatavari and its bioactive constituents have consistently demonstrated the ability to mitigate oxidative stress, enhance endogenous antioxidant defense, and suppress inflammatory mediators. Experimental evidence has consistently demonstrated that Shatavari enhances endogenous antioxidant defense systems by increasing the activity of superoxide dismutase (SOD), catalase (CAT), and glutathione peroxidase (GSH-Px) while simultaneously reducing malondialdehyde (MDA), a major biomarker of lipid peroxidation [124]. These effects are compatible with NRF2-dependent cytoprotective signaling, which can simultaneously restrain NF-κB activation and decrease the production of pro-inflammatory cytokines such as TNF-α, IL-1β, and IL-6. Through reinforcement of cellular antioxidant defenses, Shatavari may restore redox homeostasis and protect neuronal structures from oxidative damage. In accordance with these observations, our recent study has demonstrated that Shatavarin IV significantly reduced intracellular ROS levels in LPS-treated SH-SY5Y cells, further supporting its antioxidant potential [27]. Studies have also reported the neuroprotective potential of Shatavarin IV in *Caenorhabditis elegans* models of aging and Parkinsonism [35]. Treatment with Shatavarin IV in *Caenorhabditis elegans* models significantly reduced intracellular ROS accumulation, improved cholinergic neurotransmission, increased synaptic Ach levels and nAChR activity, led to the inhibition of acetyl cholinesterase activity, decreased protein carbonylation, and promoted longevity [125]. Shatavarin IV enhanced the expression of multiple antioxidant genes, including *sod-1*, *sod-2*, *sod-3*, *gst-4*, *gst-7*, and *ctl-2*, indicating robust activation of endogenous antioxidant defense mechanisms. In addition to its antioxidant effects, Shatavarin IV has been reported to alleviate Parkinsonian phenotypes by reducing α-synuclein aggregation, limiting lipid accumulation, and restoring dopamine levels. These neuroprotective effects are associated with an increased expression of genes involved in mitochondrial quality control and protein homeostasis, including *pink-1*, *pdr-1*, and *ubc-12*, indicating improved mitochondrial function, proteostasis, and ubiquitin-mediated protein degradation pathways. Together, these observations suggest that the neuroprotective actions of Shatavarin IV involve multiple cellular mechanisms and are not solely attributable to its antioxidant activity [126]. Because oxidative stress and chronic neuroinflammation profoundly influence neurotrophic signaling, the preservation of neuronal function ultimately depends on maintaining the pathways that support synaptic plasticity and neuronal survival. Among these, BDNF represents one of the most important neuroprotective mediators.

In addition to limiting oxidative damage, the preservation of cognitive function requires maintenance of neuroplasticity. Brain-derived neurotrophic factor (BDNF) is a key regulator of neuronal survival, dendritic remodeling, synaptic plasticity, learning, and memory formation. Reduced BDNF expression is frequently observed in neurodegenerative and neuropsychiatric disorders, including Alzheimer’s disease, Parkinson’s disease, depression, and age-associated cognitive decline [127]. Chronic neuroinflammation, oxidative stress, excessive ROS/RNS production, and prolonged HPA axis activation collectively contribute to the suppression of BDNF signaling, thereby impairing neuronal resilience and cognitive function [128].

Studies have suggested that Shatavari may support neurotrophic homeostasis by enhancing BDNF expression and availability. Shatavari establishes a cellular environment favorable for neurotrophic signaling through the attenuation of oxidative stress, suppression of pro-inflammatory cytokines, and stabilization of HPA axis activity [40,119]. The restoration of BDNF signaling therefore represents a plausible mechanism linking the antioxidant, anti-inflammatory, and adaptogenic properties of Shatavari to improved cognitive outcomes [40,41,108,111]. This hypothesis is supported by findings from our laboratory, where the pharmacological inhibition of TrkB using K-252a significantly attenuated the biological effects of Shatavarin IV, indicating involvement of the BDNF–TrkB signaling axis [27]. These findings suggest that Shatavarin IV may exert at least part of its neuroprotective activity through modulation of neurotrophic signaling pathways, like BDNF. Enhanced BDNF–TrkB signaling can promote synaptic maintenance, dendritic remodeling, long-term potentiation, and neuronal resilience, thereby supporting cognitive performance and protecting against neurodegenerative progression [129]. The microbiota–gut–brain axis and the preservation of redox balance are particularly important, as oxidative stress compromises intestinal barrier integrity, disrupts microbial homeostasis, and facilitates systemic inflammation that ultimately affects brain function. By limiting oxidative damage and inflammatory signaling, Shatavari may create a cellular environment that supports neuronal survival, synaptic plasticity, and BDNF-mediated neuroprotection, thereby contributing to its proposed psychobiotic potential. Nevertheless, dedicated mechanistic and clinical studies are required to establish whether NRF2 activation represents a primary molecular target underlying the neuroprotective actions of Shatavari [25].

## 8. Phytoestrogenic Support and the Estrobolome–Brain Axis

In addition to regulating neuroinflammation, oxidative stress, and neurotrophic signaling, Shatavari may influence cognitive function through endocrine mechanisms involving estrogen signaling and the gut estrobolome [99]. The roots of *Asparagus racemosus* are rich in steroidal saponins, particularly Shatavarin IV, whose structural backbone shares structural similarities with endogenous estrogens. Therefore, Shatavarin IV may modulate estrogen receptor signaling and contribute to the plant’s phytoestrogenic activity. Apart from steroidal saponins, isoflavones, which have mild estrogenic activity, also help to maintain the estrogen homeostasis [130]. Estrogen is increasingly recognized as a critical regulator of neuronal development, synaptic plasticity, neurotransmitter function and homeostasis, and behavior and mood regulation. In the CNS, estrogen exerts its effects through estrogen receptor-α (ERα), estrogen receptor-β (ERβ), and the membrane-associated G protein-coupled estrogen receptor (GPER), activating intracellular signaling pathways, such as PI3K/Akt, ERK/MAPK, and CREB, that enhance brain-derived neurotrophic factor (BDNF) expression, promote hippocampal neurogenesis, facilitate dendritic spine formation, and preserve synaptic plasticity [131]. The activation of ERα, ERβ, and GPER suppresses NF-κB-dependent inflammatory signaling, attenuates microglial activation, and reduces the production of pro-inflammatory cytokines, including TNF-α, IL-1β, and IL-6. Estrogen also enhances antioxidant defenses through functional interactions with NRF2-mediated cytoprotective pathways, thereby limiting reactive oxygen species generation, preserving mitochondrial function, and protecting neuronal viability [132]. Furthermore, estrogen modulates serotonergic, dopaminergic, and GABAergic neurotransmission by regulating neurotransmitter synthesis, receptor expression, and synaptic responsiveness. Through these coordinated mechanisms, estrogen signaling supports brain-derived neurotrophic factor (BDNF) expression, enhances neuronal resilience, and preserves cognitive function. These observations are testimonial to the close interplay between endocrine signaling, immune regulation, and the microbiota–gut–brain axis [133]. Consequently, impaired estrogen homeostasis has been associated with depression, cognitive decline, and an increased risk of neurodegenerative disorders [134].

An important component of this endocrine–microbiota interaction is the estrobolome, which comprises a collection of gut microbial genes involved in estrogen metabolism. Microbial β-glucuronidase enzymes deconjugate estrogen metabolites excreted into the intestinal lumen, allowing for free estrogens to be reabsorbed through enterohepatic circulation and thereby influencing systemic estrogen availability. Alterations in the composition of the estrobolome can modify β-glucuronidase activity, resulting in impaired estrogen recycling and disrupted endocrine homeostasis. Such changes have been linked to neuroinflammation, mood disturbances, cognitive dysfunction, and other manifestations of microbiota–gut–brain axis dysregulation that contribute to the pathophysiology of neuropsychiatric disorders [43].

The interaction between *Asparagus racemosus* (Shatavari) and the estrobolome may not be limited to its phytoestrogenic activity alone. Given its prebiotic properties, Shatavari may also influence the composition and metabolic activity of the gut microbiota, thereby affecting estrobolome functions and estrogen homeostasis. Although this relationship remains incompletely understood, the modulation of the estrobolome represents a plausible mechanism through which Shatavari could exert additional neuroendocrine benefits.

**Figure 5:**
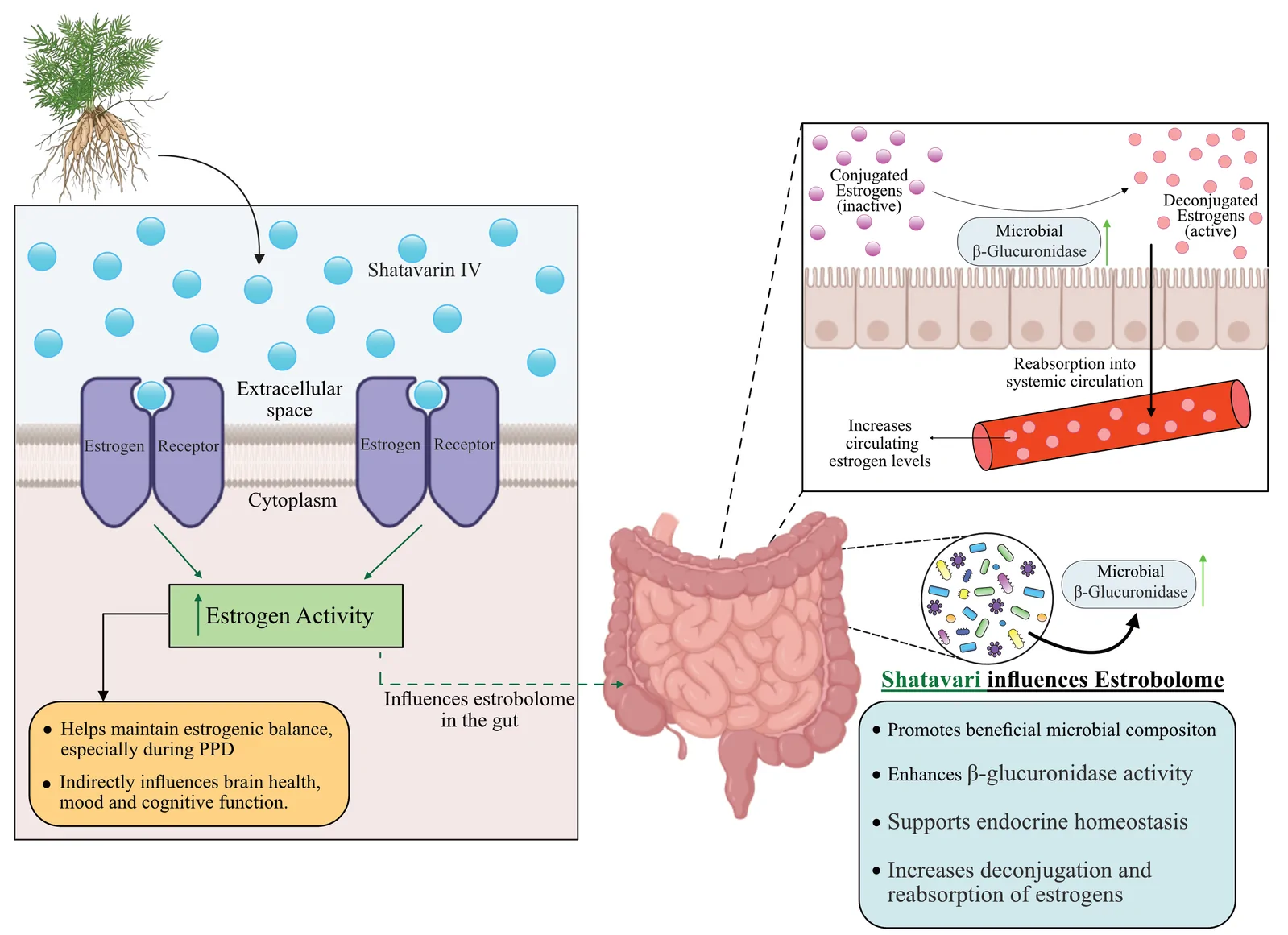
Phytoestrogenic effector mechanisms of Shatavari. The phytoestrogenic actions of *Asparagus racemosus* are exerted through estrogen receptor signaling, leading to modulation of the estrobolome, maintenance of estrogen homeostasis, and alleviation of postpartum depression (PPD).

As discussed earlier in this review, Shatavari naturally contains fructooligosaccharides (FOSs), which serve as fermentable substrates for beneficial gut microorganisms. FOSs are vital prebiotic components, and they promote microbial diversity (estrobolome) involved in estrogen metabolism. They also help maintain a balanced estrobolome and support physiological estrogen recycling. In parallel, the fermentation of FOSs generates short-chain fatty acids (SCFAs), including acetate, propionate, and butyrate, which modulate endocrine signaling and influence estrogen homeostasis through direct phytoestrogenic actions and indirect microbiota-mediated mechanisms [99] (Figure 5).

Shatavari is a renowned Ayurvedic adaptogen that has been studied extensively for its high potential to support women’s reproductive health, postpartum recovery, and management of postpartum depression (PPD) [25]. Studies of Shatavari by administering the root extracts of the plant to immature female rats have shown enhanced folliculogenesis [135], increasing the ovarian weight and elevating follicle-stimulating hormone (FSH) levels. These effects are largely attributed to the steroidal saponins, isoflavones, and other estrogenic phenolic constituents present in the plant. O’Leary et al. and the group also reported that Shatavari through it roots extracts in-creases uterine weight and glycogen content without significantly altering already circu-lating estrogen concentrations thereby highlighting that its biological effects are mediated predominantly via direct interaction with estrogen receptors rather than stimulation of endogenous estrogen synthesis [136]. Studies related to Shatavari and its influence on estrogen signaling have suggested that several interconnected molecular pathways collectively contribute to maintaining the body’s physiological homeostasis. In addition, O’Leary et al. also reported that Shatavari supplementation aided in conserving and enhancing musculoskeletal adaptation in postmenopausal women [109]. Their report suggested that the phytoestrogenic constituents of several components of Shatavari may exert systemic biological effects through modulation of estrogen receptor-mediated signaling. This receptor-mediated mechanism provides a plausible mechanistic basis for the influence of Shatavari on estrogen-dependent signaling pathways involved in neuronal plasticity, neuroinflammation, and cognitive function. However, mechanistic studies are required to establish the precise role of Shatavari in the estrobolome–brain axis and its implications in human health.

PPD remains a common mental health condition affecting 7–20% women following delivery. One of the causes of PPD is the rapid change in the reproductive hormones estradiol (the most potent and naturally occurring human estrogen) and progesterone before and immediately after delivery [10]. Symptoms include persistent sadness, severe mood swings, intense irritability, and difficulty bonding with the baby. Many experience additional symptoms including fatigue, loss of interest in activities, and, in some severe cases, recurring thoughts of harming themselves and the baby. A recent study by Ahokas et al. has shown that estrogen was effective in woman with PPD [137]. Therefore, studies suggest that estrogen treatment through hormonal therapy in woman with PPD can be effective. However, the potential benefits of hormonal therapy are often overshadowed by the magnitude of adverse effects associated with synthetic estrogens, including ethinylestradiol, estradiol valerate, and diethylstilbestrol. Shatavari contains phytoestrogens (Shatavarin IV and rutin) and thus can provide a phytopharmacological support in treating PPD. Shatavari mimics estrogen and thus can counteract the postpartum hormonal drop [25]. As Shatavari also shows prebiotic effects, which enrich the estrobolome and influence β-glucuronidase activity, this can further indirectly influence estrogen levels.

## 9. The Role of Gut Microbial Amyloids in Mediating GBA Damage

Certain gut inhabitants belonging mostly to the genera *Escherichia*, *Pseudomonas*, *Bacteroides*, and *Bacillus* are known to produce a class of biofilm-associated proteins that closely resemble human amyloids. They can form Thioflavin T- and Congo red-positive cross-beta fibrils [50] and can cross-seed each other, resulting in the development a multispecies rigid biofilm [51]. This enhances the integrity of intestinal epithelial barrier and also reinforces the mucosal homeostasis. However, the overaccumulation of such proteins may lead to their escape from the lining and their localization into the CNS, either through the vagus nerve (GBA) [52] or by a damaged blood–brain barrier. Once in the neuronal micromilieu, these proteins can seed the fibrillation of key synaptic proteins, such as α-synuclein [53]. An extensively studied class of such bacterial amyloids is the Curli, a multi-subunit key biofilm protein responsible for host colonization [55]. Curli is constituted from CsgA, a small, secreted subunit of comparable size to that of α-synuclein. CsgA polymerization is based on the nucleation subunit CsgB. The secretion of CsgA is mediated by CsgG, CsgE, and CsgF. Therefore, from a cellular perspective, CsgB or fibrillated CsgA can induce cross-seeding and nucleate fibrillation of α-synuclein. This results in exponential amplification in the number of toxic cross-beta fibrils and their subsequent deposition as complex aggregated protein entities known as amyloid plaques. The accumulation of these plaques further act as harbingers of inflammatory response, oxidative stress, and resultant degeneration of neuronal cells. Certain glycated protein adducts also result in the formation of similar amyloid-like entities [56], which explains why long-term chronic hyperglycemia or diabetes can eventually lead to cognitive impairments [57]. Therefore, it will be worthwhile to investigate the fate of the bacterial amyloids in a diabetic patient to further unravel structural clues or assists that dictate the transformation of a native folded protein into a cross-beta fibril. Apart from inducing the formation of amyloid plaques, bacterial amyloids also disrupt the immune regulation of the GBA. Microbial amyloids can serve as excellent ligands for extracellular toll-like receptor 2 (TLR-2), resulting in a generalized pro-inflammatory state in the microglial cells [50]. Thus, an altered gut microbial environment can quickly jeopardize physiological redox homeostasis and accelerate neurodegeneration and cognitive breakdown. The fermentable prebiotic fibers present in Shatavari act as excellent food sources for the gut microbial community [25]. In addition, the steroidal saponins present in the herb also enhance/replenish the gut microbial consortium. Phytotherapeutic formulations, such as the ethanolic seed extract of Fenugreek, have been known to arrest amyloid formation and propagation through a concerted synergistic action of protein and non-protein entities [58]. Similar studies with Shatavari are currently underway in our laboratory. It is pertinent here to mention that the proprietary Shatavari extract formulation SheVari4^®^ currently under investigation in our laboratory has successfully cleared the acute oral toxicity and mutagenic potential (Ames) test, and has been declared as a safe nutraceutical supplement (unpublished data).

## 10. Limitation and Future Perspectives

The mechanistic and preclinical findings summarized in this review, as well as those hypothesized from in silico analyses (summarized in Table 3), are associated with a number of shortfalls, which limit how far the results can be extrapolated in designing novel Shatavari-based therapeutics. Shatavarin IV is a glycosylated steroidal sapononin, and no study has yet measured whether it or its metabolites cross the blood–brain barrier at concentrations sufficient to impart the desired therapeutic effects. In addition, its hydrophobic skeleton, coupled with bulky sugar chains, has resulted in poor bio-absorptivity of Shatavarin-IV-based formulations. It is also pertinent here to mention that the lack of adequate pharmacokinetic data of these compounds has necessitated coupling with in silico ADMET (Absorption, Distribution, Metabolism, Excretion, and Toxicity) analyses for generating drug profiles. In this regard, the advent of Machine Learning alongside data from multi-omics pipeline have greatly facilitated the exploration of new cellular targets of Shatavari, as well as the more accurate prediction of their potential crosstalk with the bioactives from Shatavari.

## 11. Conclusions

Neuronal homeostasis is intricately associated with a healthy gut microbiota, and the constituents of Shatavari have so far demonstrated significant potentials in effectively securing this bidirectional loop. However, many of the originally proposed mechanistic insights have yet to be proved experimentally, and the routine attainment of an optimal dosage to implement these outcomes remains as an even bigger bottleneck. Current regulatory policies related to the usage of the plant differ widely across the globe In the United States, the usage of the toot extract is restricted to a nutraceutical instead of being prescribed as a full-fledged medicine with a definite structure-function claim. On the contrary, countries belonging to the EU, as well as Japan, prefer to use it as a specific botanical drug intended to augment female reproductive health instead of a dietary supplement for holistic health rejuvenation. It should be mentioned that, as a concluding note, the attainment of dosage uniformity and reproducibility will continue to be a steep terrain for Shatavari, as well as other similar medicinal herbs, until newer technologies, assisted with state-of-the-art tools and Artificial Intelligence, are adopted to completely eliminate biological contaminants from Shatavari formulations.

## Figures and Tables

**Table 1:** Natural microbial inhabitants of the gut–brain axis (GBA) and the neuromodulators produced by them.

Gut Microbial Inhabitant (Key Species)		Primary Metabolite	Mechanism of Action in the GBA	References
Genus	Key Species			
*Lactobacillus* spp.	*Lactobacillus acidophilus*, *Lactobacillus rhamonosus* and other *Lactobacillus* spp.	γ-Aminobutyric acid (GABA), dopamine, serotonin, histamine, norepinephrine, acetylcholine acetate, lactate	Upregulates glutamate decarboxylase into GABA, activates vagal inhibitory pathways. Ferments Shatavari FOSs into acetylate/lactate hence increasing butyrate synthesis and uses β-glucuronidase to activate phytoestrogenic activity.	[50,51,52,53,54]
*Bifidobacterium* spp.	*Bifidobacterium infantis*	GABA, dopamine, serotonin, histamine, norepinephrine, and acetylcholine, acetate, folate (Vit-B9)	Converts tryptophan into indole derivatives (ILA/IAA) to activate host aryl hydrocarbon receptors (AhRs), downregulating mucosal inflammation and upregulating tight-junction proteins to strengthen barrier integrity; maximizes systemic tryptophan availability to drive central serotonin biosynthesis and support downstream cognitive pathways.	[55,56,57,58,59]
*Clostridium* spp. (Cluster I)	*Clostridium butyricum*	SCFAs (butyrate, acetate), small amounts of lactate	Upregulates BDNF, suppress excessive inflammation, supports GABA-producing bacterial populations, indirectly enhances neurotransmission.	[60,61,62]
*Clostridium* spp. (Cluster XI & Cluster XIVa)	*Clostridium hiranonis*, *Clostridium scindens*	Serotonin	Produces the enzyme 7α- hydroxylase that converts primary bile acids into secondary bile acids. Secondary bile acids act as systemic signaling molecules that bind to corresponding receptors in the gut’s ECs to synthesize serotonin (5-HT).	[60,63]
*Clostridium* spp. (Cluster IV)	*Clostridium leptum*	Dopamine, norepinephrine	Produces the enzyme β-glucuronidase to cleave and deconjugate inactive host catecholamines, producing active dopamine and norepinephrine inside the gut lumen to modulate the ENS.	[54,64]
*Clostridium* spp. (Cluster XIVa)	*Clostridium symbiosum*	GABA	Upregulates glutamate decarboxylase. Synthesize GABA directly from dietary glutamate. Reduce visceral hypersensitivity in ENS and dampens the central sympathetic stress pathways.	[19,60]
*Clostridium* spp. (Cluster IV; *Ruminococcaceae*)	*Faecalibacteium prausnitzii*	Butyrate (SCFA)	Uses acetate produced from the *Lactobacillus* spp. to produce butyrate. Helps in regulating inflammatory cascade by secreting microbial anti-inflammatory molecules.	[62,65]
*Akkermansia* spp.	*Akkermansia muciniphila*	Acetate, propionate	Maintains mucosal barrier integrity, regulates immune homeostasis, and indirectly supports neuronal function through SCFA signaling.	[66,67,68]
*Bacteroides* spp.	*Bacteroides fragilis*, *Bacteroides thetaiotaomicron*, *Bacteroides vulgatus*, *Bacteroides uniformis*	GABA, acetate, propionate, sphingolipids	Influences neurotransmitter balance, modulates immune signaling, and contributes to neuronal membrane homeostasis.	[69,70,71]
*Prevotella* spp.	*Prevotella copri*, *Prevotella stercorea*, *Prevotella histicola*	Succinate, propionate	Participates in SCFA production, regulates inflammatory responses, and influences serotonin metabolism.	[62,72]
*Escherichia* spp.	*Escherichia coli*	Serotonin precursor metabolites, indole derivatives	Modulates enterochromaffin cell activity, influences serotonin availability, and activates aryl hydrocarbon receptor (AhR)-mediated immune signaling.	[57]
*Enterococcus* spp.	*Enterococcus faecalis*, *Enterococcus faecium*,	Tyramine, phenethylamine, lactate, acetate, GABA	Produces enzyme tyrosine decarboxylase. Converts dietary amino acids into trace amines like tyramine and phenethylamine. Hence, influences the enteric neuronal signaling and gastrointestinal motility. Also ferments simple carbohydrates into lactate and acetate, providing fuel for butyrate producing species, like *Faecalibacteium* spp.	[73,74]
*Bacillus* spp.	*Bacillus subtilis*, *Bacillus coagulans*, *Bacillus clausii*, *Bacillus licheniformis*	Dopamine, norepinephrine, lipopeptides (bacilomycin), SCFAs (acetate & propionate)	Contributes to catecholamine production and influences ENS activity. Aids in mucosal inflammation.	[62,65,75,76,77]

**Table 2:** Major neuroprotective bioactive constituents of *Asparagus racemosus* (Shatavari) and their proposed mechanisms of action.

Bioactive Constituent	Pharmacological Activity	Biological Activity	References
Shatavarin I	Anti-abortifacient activity (unknown)	Stabilizes the endometrium and reduces uterine hypercontractility, supporting early pregnancy maintenance (unknown).	[112]
Shatavarin IV	Antioxytocic activity, neuroprotection, immunomodulation, antiproliferative, anti-cancer activity, adaptogenic, anticandidal activity	Suppresses NF-κB-mediated inflammation, reduces oxidative stress, enhances BDNF expression, and improves neuronal survival.	[28,29,98,99,113]
Shatavarins (II, III, V-X)	Unknown	Unknown	
Sarsasapogenin	Anti-amyloidogenic	Significantly inhibits key enzymes linked to pathogenesis in Alzheimer’s disease. It inhibits pro-inflammatory cytokines, modulates HPA axis, and increases BDNF.	[98,99,100]
Polysaccharides (FOS and Inulin)	Prebiotic	Promotes growth of beneficial gut microbes, increases SCFA production, regulates the gut–brain axis, and indirectly enhances serotonergic and GABAergic signaling.	[62,98,101,114,115]
Flavonoids(Quercetin, Kaempferol, Hyperoside)	Antioxidant, anti-inflammatory	Reduces lipid peroxidation, preserves mitochondrial function, and attenuates oxidative stress-induced neuronal injury. Neutralizes ROS, activates Nrf2-dependent antioxidant pathways, inhibits pro-inflammatory cytokine production, and protects against neuronal damage.	[27,102]
Flavonoids (Isoflavones and phytoestrogenic compounds)	Estrogen-like activity	Activates estrogen receptor-mediated neuroprotective pathways, promotes synaptic plasticity, and supports BDNF signaling; particularly relevant during postpartum estrogen decline.	[24,42,69,92]
Racemofuran and Racemosal	Anti-inflammatory, antioxidant, antiparkinsonian	Inhibits inflammatory mediator production and attenuates neuroinflammatory signaling. Scavenges reactive oxygen species (ROS), protects neuronal membranes, and reduces oxidative injury.	[77,92,106,116]
Asparagamaine A	Neuroprotective alkaloid	Reduces oxidative stress and supports neuronal viability by preserving cellular redox balance.	[117]
Essential minerals (Zn, Mg, Ca, Fe, P)	Nutritional support	Serves as co-factors for neurotransmitter synthesis, antioxidant enzymes, and neuronal signaling pathways.	[98,99,101]
Vitamins (A, B_1_, B_2_, C, E)	Antioxidant	Protects neurons from oxidative damage, supports immune regulation, and preserves neuronal membrane integrity.	[99,103,104]

**Table 3:** Neuropharmacological evidence for *Asparagus racemosus* (Shatavari) and its bioactive constituents across computational, in vitro, in vivo, and clinical studies.

No.	Bioactive Molecule/Extract	Experimental Model	Dose/Concentration	Molecular Target or Pathway	Research Outcome	Ref.
A. Computational (in silico) evidence						
1	Shatavarin IV	Molecular docking	NA	GSK-3β; GSK-3–Axin complex; TNF-α; amyloid-β	Predicted concurrent binding to the tau-kinase, amyloid, and neuroinflammatory nodes; used to rationalize a brain-targeted nanoemulsion.	[121]
2	Shatavarin IV	Molecular docking	NA	TrkB (TrkB–BDNF axis)	Docks to TrkB along with BDNF rather than as a direct agonist, predicting preservation of extracellular BDNF signaling.	[27]
3	13 root phytoconstituents (Asparagamine A, Asparanin A, Shatavarins I and IV–X, sarsasapogenin, Sitosterol, Quercetin)	Network pharmacology; targets epilepsy comorbid with Alzheimer’s disease	NA	5 key molecular targets (CASP3, TNF, VEGFA, PTGS2, CNR1); 19 enriched KEGG pathways	Network- and pharmacology-based analyses revealing key therapeutic molecular targets in epilepsy comorbid with Alzheimer’s disease. Hub ligands included Shatavarins V, VI, and IX, as well as sarsasapogenin.	[138]
4	Muzanzagenin (root metabolite)	Untargeted LC-MS/MS metabolomics, with network pharmacology; comparative molecular docking	NA	BACE1	Muzanzagenin binds BACE1 with a favorable docking score supported by eight hydrogen bonds, identifying it as a predicted BACE1 inhibitor and a candidate therapeutic molecule for Alzheimer’s disease.	[139]
5	Shatavarins I, IV, and VI–X	Comparative molecular docking	NA	Estrogen receptor α, β, and γ; progesterone receptor; FSH receptor; LH receptor	Molecular docking study and analysis lead to identification of important Shatavarin derivatives and their potential roles as ligands for their corresponding female hormonal receptors.	[140]
6	Sarsasapogenin derivatives	Network-based drug–target, with structure-based docking screening	NA	20 potential target	Combining network-based and structure-based methods together to identify targets for anti-AD sarsasapogenin derivatives.	[141]
B. In vitro evidence						
7	MAR	Cell-free enzyme kinetics	MAR AChE IC_50_ 12.35 ± 1.16 mg/mL	AChE, BChE, MAO-A, and MAO-B	Competitive, non-selective inhibition of both cholinesterases and both MAO isoforms; unchanged V_max_ with rising K_m_.	[142]
8	Sarsasapogenin (SRS) and standardized aqueous extract (39.42% SRS)	Cell-free enzyme assays; Aβ42 aggregation (thioflavin T, Congo red, TEM); PC12 cells under H_2_O_2_ stress	Concentration-dependent; 44 µM for Aβ42 assay	AChE, BChE, BACE1, MAO-B; Aβ42 fibrillization; redox and apoptotic signaling	52% inhibition of Aβ42 fibrillization at 44 µM (tannic acid 85%) with fewer and shorter fibrils on TEM; 67% protection of PC12 cells against H_2_O_2_ (glutathione 91%).	[143]
9	Shatavarin IV; standardized ethanolic root extract (SheVari4^®^, 5% *w*/*v* Shatavarin IV)	LPS-stimulated SH-SY5Y cells, with and without exogenous BDNF and K252a (Trk inhibitor)	Shatavarin IV 10 ng/mL; SheVari4^®^ 100 ng/mL; LPS 1 µg/mL	TrkB–BDNF axis; cytokine and redox signaling	IL-6 ↓ ~46%, TNF-α ↓ ~50%, IL-10 ↑ ~2.74-fold, TGF-β ↑ ~4.4-fold; ROS and NO reduced. K252a abolished and exogenous BDNF augmented the effect, placing the action downstream of TrkB.	[27]
10	Aqueous root extracts	Primary rat hippocampal neuron cultures; glutamate excitotoxicity	Glutamate 100 µM for 10 min; extract co-treatment	NMDA-linked excitotoxic cascade	Preserved hippocampal neuronal viability against glutamate-induced excitotoxic damage.	[144]
11	*A. racemosus* root extract (metabolomically characterized)	Neuronal cells; ROS assay, apoptosis assay, Western blot	NR	ROS handling; apoptotic pathway proteins; BACE1 expression	Reduced ROS, altered apoptotic signaling and lowered BACE1 protein, validating the network–pharmacology predictions (from No 4 in Table 3).	[139]
12	MAR	Rats; forced swim and learned-helplessness paradigms	100, 200, 400 mg/kg p.o., 7 days	Serotonergic and noradrenergic facilitation; endogenous antioxidant defenses (dopaminergic system unaffected)	Reduced immobility in forced swim and increased avoidance responding in learned helplessness; reversed swim-stress perturbation of antioxidant enzymes.	[145]
13	MAR	Experimentally unmanipulated rats; lorazepam 0.5 mg/kg i.p. as positive control	50, 100, 200 mg/kg p.o., 7 days	Plasma corticosterone and norepinephrine; regional monoamines and turnover in hippocampus, hypothalamus, prefrontal cortex, amygdala, and nucleus accumbens	Non-suppressive modulation of the HPA axis and monoaminergic tone, with a ceiling effect at 100 mg/kg in most regions.	[146]
14	MAR	Rats; diazepam 1 mg/kg p.o. as comparator	50, 100, 200 mg/kg p.o., 7 days	GABAergic and serotonergic (5-HT_2_A, amygdala) tone with HPA modulation	100 and 200 mg/kg produced diazepam-comparable anxiolysis across rat models.	[147]
15	Ethanolic root extract	Streptozotocin-diabetic rats	100 and 200 mg/kg p.o., 7 days from week 6 of diabetes	Monoaminergic signaling, with glycemic control as a modifier	Reduced immobility, more squares crossed and rearing episodes, fewer fecal pellets; blood glucose also fell.	[148]
16	Ethanolic root extract	Mice (restraint stress, chemical writhing, and swimming endurance tests)	NR	Brain lipid peroxidation, nitric oxide, protein and glutathione; adrenal and splenic weight	Improved stress tolerance at all doses tested; normalized brain lipid peroxidation, NO and glutathione; attenuated adrenal hypertrophy and splenic atrophy.	[149]
17	Aqueous root extract, with *W. somnifera* as a comparator	Rats (unpredictable immobilization/swim stress 4 h daily for 30 days; hippocampus)	NR	Hippocampal SOD and catalase activity	Recovery of hippocampal antioxidant enzyme activity after chronic stress.	[150]
18	MAR	Rats (Morris water maze and elevated plus maze; scopolamine- and sodium-nitrite-induced amnesia)	50, 100, 200 mg/kg p.o., 7 days	Dose-dependent AChE inhibition in prefrontal cortex, hippocampus and hypothalamus	Shorter escape latency in the Morris water maze; reversal of scopolamine- and NaNO_2_-induced increase in transfer latency.	[151]
19	Root extract	Scopolamine-amnesic mice (hippocampal histology)	NR	Cholinergic signaling; hippocampal neuronal integrity	Preserved hippocampal neurons with improved memory performance.	[152,153]
20	Ethanolic root extract	Ovariectomized Wistar rats	100 and 1000 mg/kg/day by gavage	Increased BDNF with upregulation of ERα and ERβ	Reversed ovariectomy-induced loss of BDNF and estrogen receptors in frontal cortex and hippocampus.	[154]
21	Ethanolic root extract	Ovariectomized Wistar rats	100 and 1000 mg/kg/day	Serum estradiol; regional neuronal viability	Improved recognition memory with higher neuronal viability in both regions.	[155]
22	Root extract	Swiss albino mice (unpredictable swim stress 3 h/day for 30 days; open field and passive avoidance; hippocampal cytology)	100 mg/kg p.o., 30 days	Region-specific hippocampal neurodegeneration (CA1, CA3, CA4, dentate gyrus)	Cell counts in hippocampal subfields restored towards control; degeneration ranked CA3 > DG > CA1 > CA4; protection ranked DG > CA4 > CA1 > CA3.	[156]
23	Root extract	Pentylenetetrazole kindling in rats (35 mg/kg i.p. every 48 ± 2 h, 21 injections over 43 days); valproate 300 mg/kg i.p.; forced swim and step-down passive avoidance	NR	Regional GABA, glutamate and monoamine changes in discrete brain regions	Suppressed kindling progression and lowered seizure severity score; reduced immobility and increased step-down latency, correcting the kindling-associated depression-like behavior and memory deficits.	[157]
24	Root extract	Mouse catamenial epilepsy model (cortex and hippocampus analyzed)	200, 400, 800 mg/kg i.p., 10 days	Progesterone, estradiol and corticosterone; GAD activity; monoamine levels	Reduced seizure susceptibility and depression-like behavior after neurosteroid withdrawal.	[158]
25	Ethanolic root extract	Maximal electroshock in Wistar rats and pentylenetetrazole seizures in mice	100, 200, 400 mg/kg p.o.	GABAergic mechanisms	200 and 400 mg/kg abolished the extensor phase, protecting 33.34% and 66.67% of rats respectively; 400 mg/kg prolonged postictal depression.	[159]
26	Methanolic root extract	Maximal electroshock and pentylenetetrazole (90 mg/kg) seizure models in rats	200 mg/kg p.o.	Not resolved	Methanolic extract gave the shortest hind-limb extension (~7 s vs. ~14 s in controls) and the longest delay to convulsion onset (~495 s vs. ~147 s).	[160]
27	Ethanolic root extract, compared with methanolic	Maximal electroshock model in rodents, with phytochemical characterization	500 mg/kg p.o.	Not resolved	Significant reduction in hind-limb extension, with the ethanolic extract outperforming the methanolic.	[161]
28	Methanolic root extract	Global cerebral ischemia in Wistar rats (bilateral carotid occlusion, 15 min, with reperfusion)	200 and 400 mg/kg, 7 days before and 7 days after induction	SOD, glutathione peroxidase, glutathione reductase, catalase; lipid peroxidation; AChE; glutamate, dopamine, and serotonin	Cerebroprotection with restored antioxidant and neurotransmitter profiles and improved brain histopathology (*p* < 0.01).	[162]
29	Hydroalcoholic extracts of *Camellia sinensis*, *A. racemosus*, and *Mucuna pruriens*, alone and in combination	Haloperidol-induced catalepsy rat model of Parkinsonism	NR	Dopaminergic signaling and brain antioxidant enzymes	Antiparkinsonian and antioxidant effects, with the combination outperforming single extracts.	[163]
30	Polyherbal formulation containing *A. racemosus*, *Convolvulus prostratus*, *Bacopa monnieri*, and *Nigella sativa*	Haloperidol catalepsy screen and DPPH assay; HPTLC; docking of marker actives	NR	Dopaminergic and antioxidant pathways	*A. racemosus* was one of four extracts selected on efficacy from an initial panel of seven for formulation development.	[164]
31	Integrated extract of *Trigonella foenum-graecum* (TFG) and *A. racemosus* (AR) roots	Rats were randomly divided into nine groups 4 weeks after ovariectomy	Combination of TFG and AR (both ranging from 15 to 40 mg/kg BW); combination of both at 30 and 60 mg/kg BW	FSH, LH, cortisol, estradiol, progesterone, dopamine; c-FOS, GnRH, kisspeptin, neurokinin B, TRPV1; BDNF; MDA and antioxidant enzymes	FSH, LH, and cortisol fell while estradiol, progesterone, and dopamine rose (*p* < 0.0001); hypothalamic KNDy markers and TRPV1 fell and BDNF was restored, alleviating hot-flash-like symptoms.	[165]
C. Clinical evidence						
32	Shatavari root extract	Randomized, double-blind, placebo-controlled trial; perimenopausal women aged 40–55 y; 80 randomized, 73 completed per protocol	300 mg once daily, 8 weeks	Phytoestrogenic modulation; HPA and perceived-stress axis; serum estradiol	Total Menopause Rating Scale fell 42.6% from baseline versus 5.5% on placebo; perceived stress fell 29.6% and hot-flash score 25.9% versus ~7%; POMS fatigue improved (*p* = 0.019); serum estradiol rose 32.8%. No adverse events.	[166]
33	Root extract	Randomized, double-blind, placebo-controlled trial; healthy perimenopausal women aged 38–54 y, India; 150 randomized (ITT), 145 completed	500 mg/day, 84 days	Phytoestrogenic receptor binding in mood-regulating regions, as proposed by the authors	Primary endpoint (MSSI-38) improved, with secondary gains across psychological, cognitive, sleep, stress, fatigue, sexual, and quality-of-life domains; cognitive, mood, and vitality gains emerged from day 28.	[167]
34	Root extract	Randomized, double-blind, placebo-controlled trial in women with polycystic ovary syndrome	NR; 12 weeks	Hypothalamic–pituitary–ovarian axis; perceived stress	Perceived Stress Scale fell 19.05%; ovarian volume fell 17.72%; follicle count fell 28.6%; endometrial thickness rose 25.98%; mild-to-moderate reductions in FSH, LH, estradiol, testosterone, progesterone and DHEA-S.	[135]
35	Root extract	Open-label clinical arm accompanying the murine stress study in row 22; patients with cognitive disorders at an Ayurvedic hospital	NR	Phytoestrogen-mediated CNS activity, as proposed by the authors	Improvement in memory retention and recall, paralleling the hippocampal protection seen in the animal arm.	[156]
36	Root extract	Multi-center, randomized, double-blind, parallel-group trial; 160 perimenopausal women (India and USA)	300 mg/day, 12 weeks	Adaptogenic and phytoestrogenic mechanisms; physiological stress markers	Protocol only. Primary endpoint was the Menopause Rating Scale; secondary endpoints cover mood, sleep, and stress questionnaires and physiological stress markers.	[168]

The up and down arrows indicate increase and decrease respectively.

## Data Availability

No new data were created or analyzed in this study. Data sharing is not applicable to this article.
